# Associations between Life’s Essential 8 and liver function: a cross-sectional study

**DOI:** 10.3389/fnut.2024.1515883

**Published:** 2025-01-06

**Authors:** Qiaoli Liang, Menglong Zou, Ziming Peng

**Affiliations:** ^1^Doumen Qiaoli Hospital of Traditional Chinese Medicine, Zhuhai, Guangdong, China; ^2^The First Hospital of Hunan University of Traditional Chinese Medicine, Changsha, Hunan, China; ^3^Fangchenggang Hospital of Traditional Chinese Medicine, Fangchenggang, Guangxi, China

**Keywords:** Life’s Essential 8, NHANES, liver function, cardiovascular health, cross-sectional study

## Abstract

**Background:**

Life’s Essential 8 (LE8) score, developed by the American Heart Association, assesses cardiovascular health using eight components: diet, physical activity, nicotine exposure, sleep health, body mass index, lipids, blood glucose, and blood pressure. Liver function is a critical indicator of overall health, with impairments linked to numerous chronic diseases. While the LE8 score has been extensively studied in relation to cardiovascular outcomes, its association with liver function remains underexplored. Understanding this relationship is crucial for integrating cardiovascular and hepatic health management, particularly given the shared metabolic pathways underlying these systems. This study aims to examine the relationship between LE8 scores and liver function indicators in a large cohort, addressing a critical gap in understanding the interplay between cardiovascular and liver health.

**Methods:**

Data from the 2007–2018 National Health and Nutrition Examination Survey (NHANES) were used in this cross-sectional study. The study included 21,873 participants, stratified into low (0–49), moderate (50–79), and high (80–100) LE8 score categories. The relationship between LE8 scores and liver function markers, including alanine aminotransferase (ALT), aspartate aminotransferase (AST), alkaline phosphatase (ALP), gamma-glutamyl transferase (GGT), and ALT/AST ratio, was evaluated using multivariable linear regression, smoothed curve fitting, threshold effect analysis, and weighted quantile sum (WQS) regression. Subgroup analyses were performed based on sex and age to assess potential interactions.

**Results:**

Higher LE8 scores were significantly associated with improved liver function, particularly highlighted by two major findings. First, nonlinear associations were observed between LE8 scores and liver function parameters, including ALT and ALT/AST ratio, with stronger effects beyond specific thresholds (ALT: 50.625, ALT/AST: 61.875). Second, subgroup analyses revealed that these associations were more pronounced in younger participants (<60 years), suggesting age-specific differences in the relationship. These age-related differences might be attributed to variations in metabolic function or differences in the severity of cardiovascular and liver-related risk factors between younger and older individuals. WQS regression identified body mass index, blood pressure, blood glucose, and nicotine exposure as the strongest contributors to liver function markers. These findings underscore the potential of LE8 scores as a comprehensive indicator for liver health, particularly in younger populations.

**Conclusion:**

This study suggests that LE8 scores is associated with improved liver function. Clinicians and public health practitioners could consider integrating LE8 scores into routine assessments to help identify individuals at risk for liver dysfunction, particularly among younger populations. Further research should explore whether interventions targeting cardiovascular health could also improve liver function outcomes.

## 1 Introduction

The liver, a vital organ responsible for metabolism, detoxification, and biochemical synthesis, is essential for maintaining overall health (1). Approximately 2 million deaths occur each year due to liver diseases (2). Liver function can be impaired by various factors such as viral infections, excessive alcohol consumption, drug-induced hepatotoxicity, and metabolic disorders (3). Liver function parameters such as alanine aminotransferase (ALT), aspartate aminotransferase (AST), the ALT/AST ratio, gamma-glutamyl transferase (GGT), and alkaline phosphatase (ALP) are essential indicators for assessing liver health. Moreover, they are involved in metabolic processes that link liver health to other bodily systems. For example, within the Framingham Heart Study cohort, higher GGT levels were associated with increased plasma triglycerides, body mass index (BMI), and blood pressure (4). Given these connections, the relationship between liver function and cardiovascular health (CVH) has attracted increasing attention.

In 2010, the American Heart Association (AHA) introduced Life’s Simple 7 (LS7), a set of metrics for assessing CVH (5). However, the LS7 did not account for individual variations and changes over time, prompting the AHA to develop Life’s Essential 8 (LE8) in 2022 (6). The LE8 score includes eight key measures: diet, physical activity, nicotine exposure, sleep health, BMI, lipids, blood glucose, and blood pressure. The LE8 score has shown promise in predicting a range of health outcomes beyond CVD. Higher LE8 scores are inversely associated with several non-communicable diseases, including biological aging (7), testosterone deficiency (8), and depression (9), and is associated with increased longevity (10). Emerging evidence also suggests a connection between CVH, as measured by LE8, and liver diseases (11, 12). It is worth noting that the components of LE8 are not only important for CVH, but also have potential effects on liver function. For instance, poor sleep health has been associated with metabolic dysregulation, which can lead to liver fat accumulation and increased liver enzymes. Nicotine exposure has been linked to oxidative stress, which may contribute to liver injury and inflammation. Elevated blood glucose levels are a known risk factor for non-alcoholic fatty liver disease (NAFLD), which in turn can elevate liver enzymes such as ALT and AST. Similarly, high BMI and poor lipid profiles are associated with liver fat deposition and hepatocyte damage, potentially increasing liver enzyme levels. Given these associations, the LE8 score may be an effective tool for assessing overall liver function. While some studies have shown associations between poor CVH and adverse liver outcomes (13–15), few have explored the role of comprehensive CVH measures like LE8 in relation to specific liver function parameters. In addition, most studies assume a linear relationship between CVH and liver outcomes without considering potential non-linear associations.

The National Health and Nutrition Examination Survey (NHANES) is a nationally representative dataset. NHANES includes detailed demographic, lifestyle, and clinical data, making it ideal for examining the association between LE8 scores and liver function. The purpose of this study is to examine the association between LE8 scores and liver function parameters in a representative sample of US adults. Additionally, through nonlinear curve fitting and subgroup analysis, we aim to reveal complex, age-dependent associations between CVH and liver function, providing novel insights into how improving CVH might protect liver function.

## 2 Materials and methods

### 2.1 Study population

This study utilized data from the NHANES spanning the years 2007 to 2018. NHANES was approved by the National Center for Health Statistics (NCHS) Ethics Review Board, and all the participants provided written informed consent. The research was conducted in accordance with the STROBE reporting criteria for cross-sectional studies.

**Figure 1:**
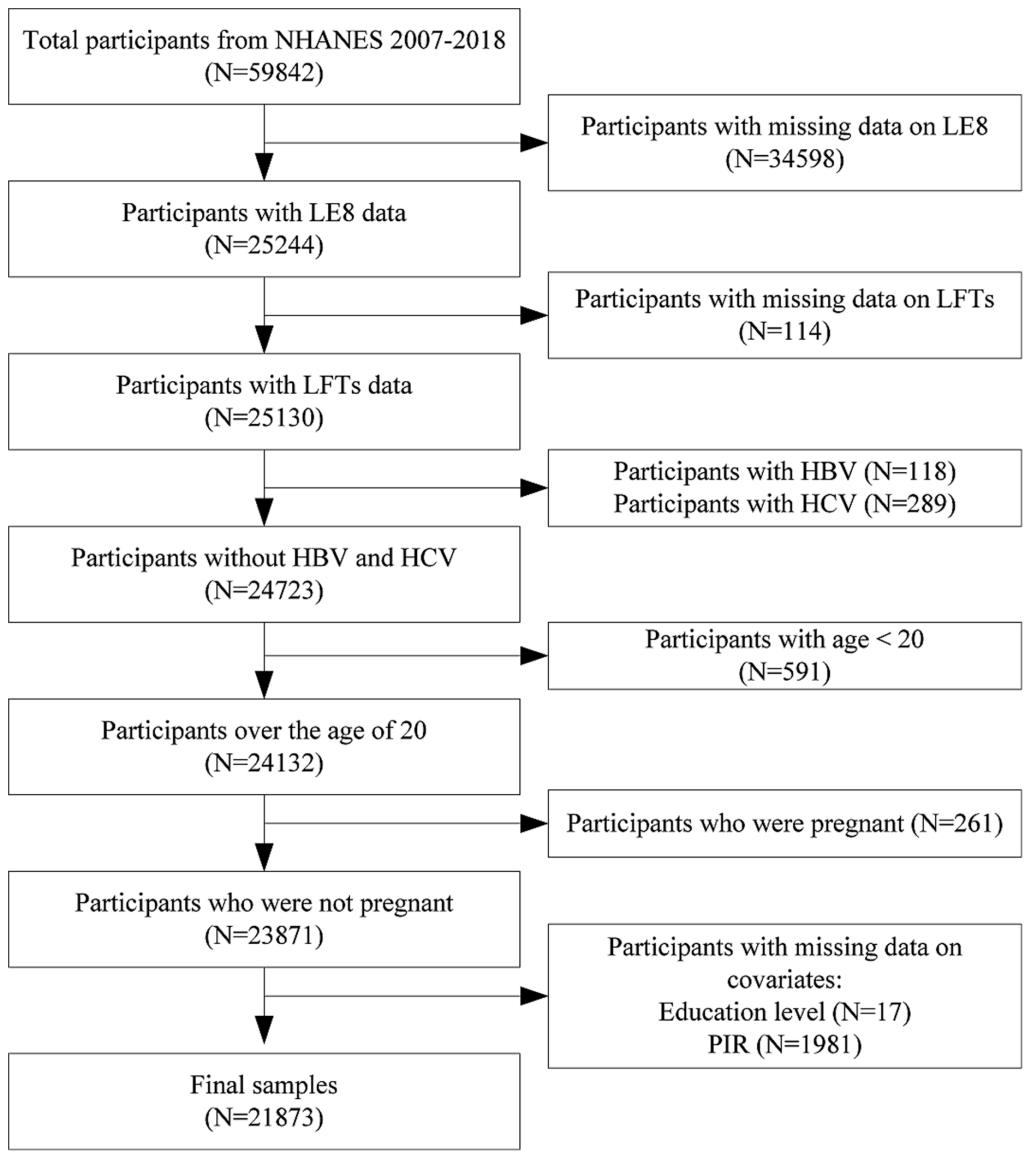
Flowchart of participants selection. NHANES, national health and nutrition examination survey; LE8, life’s Essential 8; LFTs, liver function tests; HBV, hepatitis B virus; HCV, hepatitis C virus.

Initially, 59,842 participants were included in the dataset. Participants were excluded for the following reasons: 34,598 for missing LE8 data, 114 for missing liver function data, 118 for being hepatitis B surface antigen positive, 289 for being hepatitis C RNA positive, 591 for being younger than 20 years, 261 for being pregnant, and 1,998 for missing covariate data (17 for education level, 1,981 for family poverty income ratio). Ultimately, the study included 21,873 participants. A detailed participant flow diagram is provided in Figure 1 to visually represent the exclusion process.

### 2.2 LE8 scoring

The LE8 score includes eight CVH indicators: four health factors (BMI, blood pressure, blood glucose, non-high-density lipoprotein cholesterol (HDL)) and four health behaviors (diet, nicotine exposure, physical activity, sleep health). Diet metric was assessed using the Healthy Eating Index-2015 (HEI-2015), which is based on two 24-h dietary recall interviews. The HEI-2015 score is a measure of adherence to dietary guidelines and overall diet quality. Physical activity was measured by self-reported questionnaires on the frequency and duration of vigorous or moderate-intensity physical activity per week. Secondhand smoke exposure and self-reported smoking status were used to determine nicotine exposure. The assessment of sleep health was done through self-reported average sleep duration each night. BMI was calculated from measured weight and height (kg/m^2^). To determine blood pressure, three consecutive readings were averaged during the physical exam. The measurement of blood glucose was done using fasting blood glucose or glycated hemoglobin (HbA1c) from blood samples. HDL cholesterol was calculated from blood lipid profiles. Each indicator is scored on a scale from 0 to 100, and the overall LE8 score is calculated as the mean of these eight scores (Supplementary Table S1). A score of 80–100 denotes high CVH, 50–79 indicates moderate CVH, and 0–49 reflects low CVH.

### 2.3 Liver function assessment

Fasting blood samples were collected at NHANES mobile examination centers and analyzed centrally using the Beckman Coulter DxC800 Synchron clinical system. Liver function parameters include ALT, AST, GGT, ALP, and the ALT/AST ratio. These parameters help measure liver function and detect liver damage. ALT is an enzyme found primarily in the liver that is critical for amino acid metabolism. Elevated ALT levels indicate liver cell damage and can be an early marker of liver disease. AST is found in the liver, muscles, heart, and other tissues. Although not as specific to the liver as ALT, increased AST levels also suggest liver injury or damage. The ALT/ AST ratio helps to differentiate between various liver diseases. For example, a ratio greater than 1 typically indicates alcoholic liver disease, while a ratio less than 1 indicates non-alcoholic fatty liver disease or chronic hepatitis. ALP is related to the bile ducts. Elevated ALP levels may indicate bile duct obstruction, cholestasis, or other liver disease. GGT is involved in the metabolism of glutathione and is an indicator of liver disease, particularly those involving cholestasis or bile duct obstruction.

### 2.4 Measurement of covariates

Given the large number of variables in the LE8 score, this study adjusted for a limited number of covariates to avoid overfitting the model. The covariates included in this study were age, gender, race, education level, and poverty income ratio (PIR).

### 2.5 Statistical analysis

To account for the complex sampling design of the NHANES data, weighted analyses were conducted according to NCHS guidelines. Weights, strata, and primary sampling units were considered in this study. Continuous variables were expressed as weighted means and compared using weighted linear regression. Categorical variables were presented as counts (weighted percentages) and compared using weighted chi-square tests. The association between LE8 scores and liver function biomarkers was evaluated using weighted univariate and multivariate linear regression models. Model 1 evaluated the raw relationship between LE8 score and liver function without covariate adjustment. Model 2 adjusted for gender, age, and race. Model 3 further adjusted for education level and PIR based on Model 2. Dose–response relationships were examined using smoothed curve fitting, and weighted quantile sum (WQS) regression models were used to analyze the relationships between mixed exposures of LE8 indicators and liver function, as well as the relative contributions of each indicator. A *p*-value of less than 0.05 (two-sided) was defined as statistically significant. Statistical analyses were performed using EmpowerStats (version 4.2) and R software (version 4.3.0).

## 3 Results

### 3.1 Baseline characteristics

Baseline characteristics of the study population, stratified by LE8 score category, are detailed in Table 1. A total of 21,873 participants were included, divided into low (*N* = 4,149), moderate (*N* = 15,177), and high (*N* = 2,547) LE8 score groups. The mean age showed a decreasing trend across the LE8 score groups, with the highest age observed in the low LE8 score group (53.22 years, 95% CI: 52.52–53.93), followed by the moderate (48.05 years, 95% CI: 47.45–48.65), and the lowest in the high score group (39.77 years, 95% CI, 38.78–40.76), with significant differences between groups (*p* < 0.001). Gender distribution also varied across LE8 score groups, with a higher proportion of females in the high LE8 score group (59.45%) compared to the low (51.53%) and moderate (50.03%) groups (*p* < 0.001). Racial composition differed significantly across the LE8 score categories, with non-Hispanic white participants more prevalent in the high LE8 score group (73.85%) compared to the low (62.52%) and moderate (68.65%) groups, while non-Hispanic black participants were more concentrated in the low score group (17.14%) (*p* < 0.001). Educational attainment showed a clear gradient with increasing LE8 score. A higher proportion of participants with education above high school was observed in the high LE8 score group (80.44%) compared to the low (46.54%) and moderate (63.51%) groups (*p* < 0.001). Similarly, PIR demonstrated significant differences, with the high LE8 score group showing a larger percentage of participants in the highest income category (PIR >3: 59.26%) compared to the low (34.95%) and moderate (51.94%) groups (*p* < 0.001). Liver function parameters, including ALT, AST, ALT/AST ratio, ALP, and GGT levels, displayed significant and consistent decreases across the increasing LE8 score categories (all *p* < 0.001), with the lowest levels seen in the high LE8 score group.

**Table 1:** Baseline characteristics of participants according to LE8 score.

Characteristics	LE8 score			*P*-value
	Low (*N* = 4,149)	Moderate (*N* = 15,177)	High (*N* = 2,547)	
Age, years	53.22 (52.52–53.93)	48.05 (47.45–48.65)	39.77 (38.78–40.76)	<0.001
Gender				<0.001
Male	2003 (48.47%)	7,527 (49.97%)	1,020 (40.55%)	
Female	2,146 (51.53%)	7,650 (50.03%)	1,527 (59.45%)	
Race				<0.001
Mexican American	594 (8.62%)	2,195 (8.61%)	358 (7.45%)	
Other Hispanic	389 (5.5%)	1,510 (5.48%)	254 (5.45%)	
Non-Hispanic White	1705 (62.52%)	6,938 (68.65%)	1,258 (73.85%)	
Non-Hispanic Black	1,214 (17.14%)	2,877 (9.6%)	258 (5.01%)	
Other Race-Including Multi-Racial	247 (6.21%)	1,657 (7.66%)	419 (8.25%)	
Education level				<0.001
Less than high school	1,317 (24.4%)	3,116 (13.21%)	286 (6.49%)	
Completed high school	1,093 (29.06%)	3,495 (23.27%)	381 (13.06%)	
Above high school	1739 (46.54%)	8,566 (63.51%)	1880 (80.44%)	
PIR				<0.001
≤1.3	1737 (33.12%)	4,362 (19.82%)	583 (15.89%)	
1.3–3	1,366 (31.93%)	4,847 (28.25%)	720 (24.84%)	
>3	1,046 (34.95%)	5,968 (51.94%)	1,244 (59.26%)	
ALT, U/L	28.10 (27.20–29.00)	24.95 (24.59–25.30)	20.90 (20.20–21.61)	<0.001
AST, U/L	26.27 (25.43–27.11)	24.74 (24.45–25.03)	24.29 (23.37–25.21)	0.001
ALT/AST	1.05 (1.04–1.07)	0.99 (0.98–1.00)	0.87 (0.85–0.88)	<0.001
ALP, U/L	76.22 (75.06–77.38)	67.70 (67.10–68.29)	60.10 (58.92–61.27)	<0.001
GGT, U/L	38.04 (35.72–40.36)	26.68 (25.82–27.53)	18.66 (16.52–20.80)	<0.001

For continuous variables: survey-weighted mean (95% CI), *p*-value was by survey-weighted linear regression. For categorical variables: survey-weighted N (percentage), *p*-value was by survey-weighted Chi-square test. LE8, Life’s Essential 8; PIR, family income-to-poverty ratio; ALT, alanine aminotransferase; AST, aspartate aminotransferase; GGT, gamma-glutamyl transferase; ALP, alkaline phosphatase.

### 3.2 Relationship LE8 score and liver function parameters

Analysis revealed significant negative associations between LE8 scores and liver function indicators in all models (Table 2). For ALT, each one-point increase in the LE8 score was associated with a corresponding decrease in ALT levels, with *β*-values of −0.164 (95% CI: −0.187, −0.141) in Model 1, −0.196 (95% CI: −0.219, −0.172) in Model 2, and − 0.214 (95% CI: −0.239, −0.189) in Model 3 (all *p* < 0.001). Similarly, participants in the moderate (50–79) and high (80–100) LE8 categories had significantly lower ALT levels compared to the low (0–49) LE8 group. Similar trends were observed for AST, where each one-point increase in LE8 score corresponded to decreases in AST levels, with *β*-values of −0.054 (95% CI: −0.076, −0.032) in Model 1, −0.051 (95% CI: −0.073, −0.028) in Model 2, and − 0.057 (95% CI: −0.082, −0.031) in Model 3 (all *p* < 0.001). The ALT/AST ratio, ALP and GGT also showed consistent negative associations with LE8 scores, suggesting that higher LE8 scores are associated with better liver function. Specifically, for each one-point increase in LE8 score, ALP levels declined by 0.369 U/L in Model 1, 0.320 U/L in Model 2, and 0.271 U/L in Model 3 (all *p* < 0.001). GGT levels also decreased significantly with each one-point increase in LE8 score, with *β*-values of −0.472 in Model 1, −0.458 in Model 2, and −0.463 in Model 3 (all *p* < 0.001). These results consistently suggest that better CVH, as measured by LE8 scores, is associated with lower levels of liver enzymes.

**Table 2:** Association between LE8 and liver function parameters.

	Model 1	Model 2	Model 3
	β (95%CI) *p* value	β (95%CI) *p* value	β (95%CI) *p* value
ALT			
Life’s Essential 8 (per 1 points)	−0.164 (−0.187, −0.141) <0.001	−0.196 (−0.219, −0.172) <0.001	−0.214 (−0.239, −0.189) <0.001
LE8 classification			
Low (0–49)	Ref	Ref	Ref
Moderate (50–79)	−3.151 (−4.051, −2.251) <0.001	−4.054 (−4.921, −3.188) <0.001	−4.432 (−5.321, −3.544) <0.001
High (80–100)	−7.195 (−8.410, −5.979) <0.001	−8.204 (−9.413, −6.995) <0.001	−8.723 (−9.940, −7.506) <0.001
AST			
Life’s Essential 8 (per 1 points)	−0.054 (−0.076, −0.032) <0.001	−0.051 (−0.073, −0.028) <0.001	−0.057 (−0.082, −0.031) <0.001
LE8 classification			
Low (0–49)	Ref	Ref	Ref
Moderate (50–79)	−1.530 (−2.382, −0.677) <0.001	−1.619 (−2.491, −0.747) <0.001	−1.734 (−2.645, −0.822) <0.001
High (80–100)	−1.980 (−3.193, −0.768) 0.002	−1.654 (−2.882, −0.425) <0.001	−1.805 (−3.124, −0.485) 0.009
ALT/AST			
Life’s Essential 8 (per 1 points)	−0.004 (−0.004, −0.004) <0.001	−0.005 (−0.006, −0.005) <0.001	−0.006 (−0.006, −0.005) <0.001
LE8 classification			
Low (0–49)	Ref	Ref	Ref
Moderate (50–79)	−0.061 (−0.076, −0.046) <0.001	−0.088 (−0.103, −0.073) <0.001	−0.099 (−0.114, −0.083) <0.001
High (80–100)	−0.186 (−0.208, −0.163) <0.001	−0.228 (−0.250, −0.206) <0.001	−0.242 (−0.265, −0.220) <0.001
ALP			
Life’s Essential 8 (per 1 points)	−0.369 (−0.396, −0.341) <0.001	−0.320 (−0.351, −0.290) <0.001	−0.271 (−0.300, −0.241) <0.001
LE8 classification			
Low (0–49)	Ref	Ref	Ref
Moderate (50–79)	−8.522 (−9.746, −7.298) <0.001	−7.504 (−8.760, −6.247) <0.001	−6.288 (−7.503, −5.072) <0.001
High (80–100)	−16.123 (−17.605, −14.641) <0.001	−13.545 (−15.033, −12.057) <0.001	−11.150 (−12.603, −9.697) <0.001
GGT			
Life’s Essential 8 (per 1 points)	−0.472 (−0.527, −0.416) <0.001	−0.458 (−0.521, −0.396) <0.001	−0.463 (−0.530, −0.396) <0.001
LE8 classification			
Low (0–49)	Ref	Ref	Ref
Moderate (50–79)	−11.362 (−13.870, −8.853) <0.001	−11.154 (−13.858, −8.450) <0.001	−11.044 (−13.688, −8.400) <0.001
High (80–100)	−19.382 (−22.598, −16.167) <0.001	−17.930 (−21.292, −14.567) <0.001	−17.539 (−21.002, −14.075) <0.001

Model 1: Adjusted for no covariates. Model 2: Adjusted for age, gender, and race. Model 2: Adjusted for age, gender, race, education and RIP (ratio of family income to poverty). LE8, Life’s Essential 8; ALT, alanine aminotransferase; AST, aspartate aminotransferase; GGT, gamma-glutamyl transferase; ALP, alkaline phosphatase.

### 3.3 Smoothed curve and threshold effect analysis

**Figure 2:**
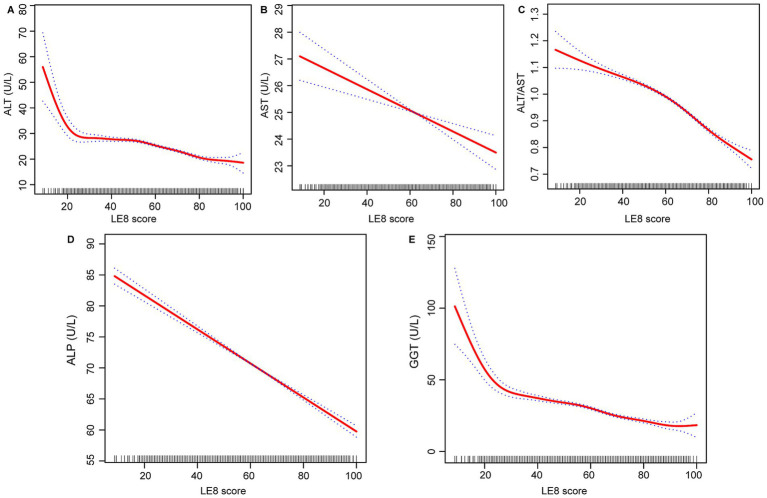
Relationship between LE8 score, **(A)** ALT, **(B)** AST, **(C)** ALT/AST, **(D)** ALP, and **(E)** GGT. LE8, life’s Essential 8; ALT, alanine aminotransferase; AST, aspartate aminotransferase; GGT, gamma-glutamyl transferase; ALP, alkaline phosphatase.

The effect relationship between LE8 score and liver function parameters was shown by smooth curve fitting (Figures 2A–E). The dose–response relationship was further assessed by threshold effect analysis (Table 3), which revealed significant nonlinear associations (*p*-value <0.001 for log-likelihood ratio test) between LE8 score and two liver function parameters (ALT and ALT/AST ratio), while the nonlinear effects for the other three liver function parameters (AST, ALP, and GGT) were not significant (*p*-values for log-likelihood ratio test of 0.378, 0.190, 0.059, respectively). For ALT, we identified an inflection point at 50.625. To the left of this inflection point, the estimated effect for ALT was −0.079 (95% CI: −0.137, −0.021, *p* = 0.008), whereas to the right, the effect increased significantly to −0.211 (95% CI: −0.236, −0.187, *p* < 0.001). This difference in effect between the two segments was significant (−0.132, 95% CI: −0.204, −0.061, *p* < 0.001). This suggests that the negative correlation between ALT and LE8 scores is stronger when LE8 scores are greater than 50.625, with ALT levels decreasing by 0.211 U/L for each 1-point increase in LE8 scores. The inflection point for the association of LE8 scores with ALT/AST ratio was 61.875, with an estimated effect of −0.004 on the left side of the inflection point, increasing to −0.007 on the right side of the inflection point. Similarly, AST, ALP, and GGT showed some variation in effects before and after their respective inflection points. However, the difference in AST and ALP did not reach statistical significance (*p* = 0.378 for AST, *p* = 0.190 for ALP), while GGT showed borderline significance (*p* = 0.059).

**Table 3:** Threshold effect analysis of LE8 on liver function parameters.

LE8 scores	Model: threshold effect analysis [*β* (95% CI) *p* value]				
	ALT	AST	ALP	GGT	ALT/AST
Inflection point (K)	50.625	50.625	84.375	38.75	61.875
<K, effect 1	−0.079 (−0.137, −0.021) 0.008	−0.017 (−0.070, 0.036) 0.529	−0.281 (−0.307, −0.255) <0.001	−0.714 (−1.021, −0.408) <0.001	−0.004 (−0.004, −0.003) <0.001
>K, effect 2	−0.211 (−0.236, −0.187) <0.001	−0.046 (−0.068, −0.024) <0.001	−0.118 (−0.353, 0.118) 0.328	−0.403 (−0.448, −0.358) <0.001	−0.007 (−0.007, −0.006) <0.001
Difference between the effects of 2 and 1	−0.132 (−0.204, −0.061) <0.001	−0.029 (−0.094, 0.036) 0.378	0.163 (−0.081, 0.407) 0.190	0.311 (−0.012, 0.634) 0.059	−0.003 (−0.004, −0.002) <0.001
Log-likelihood ratio	<0.001	0.378	0.190	0.059	<0.001

Age, gender, race, education and RIP (ratio of family income to poverty) were adjusted. LE8, Life’s Essential 8; ALT, alanine aminotransferase; AST, aspartate aminotransferase; GGT, gamma-glutamyl transferase; ALP, alkaline phosphatase.

### 3.4 WQS regression

**Figure 3:**
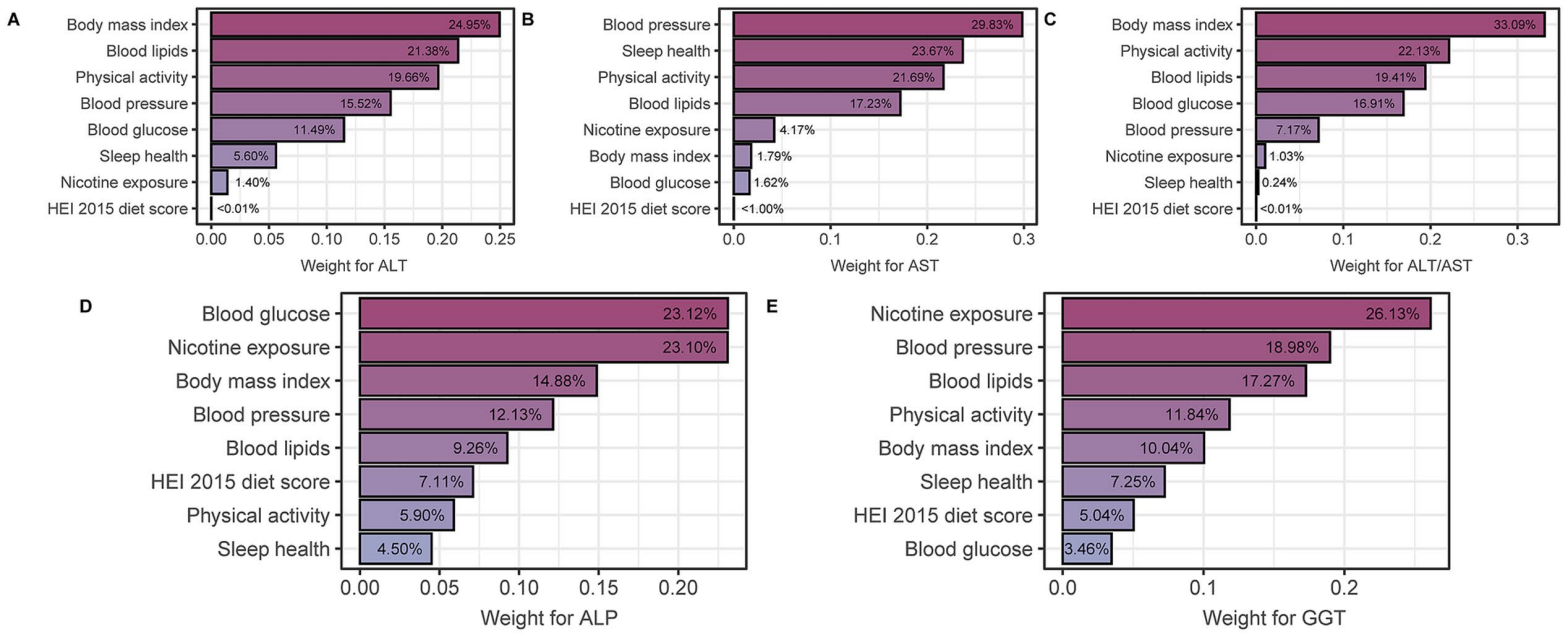
WQS model regression index weights for **(A)** ALT, **(B)** AST, **(C)** ALT/AST, **(D)** ALP, and **(E)** GGT, adjusted for age, gender, race, education and RIP (ratio of family income to poverty). LE8, life’s Essential 8; ALT, alanine aminotransferase; AST, aspartate aminotransferase; GGT, gamma-glutamyl transferase; ALP, alkaline phosphatase.

The eight components that make up the LE8 score were evaluated for their impact on these liver function parameters (Figures 3A–E). Specifically, WQS regression analyses were performed to assess the relative contribution of different components to different liver function parameters. For ALT, BMI and blood lipids were identified as the most influential factors with weights of 24.95 and 21.38%, respectively. Similarly, blood pressure and sleep health emerged as the most significant contributors to AST, accounting for 29.83 and 23.67%, respectively. For the ALT/AST ratio, BMI and physical activity were important determinants with weights of 33.09 and 22.13%, respectively. Blood glucose had the greatest contribution to ALP with a weight of 23.12%, while nicotine exposure had the greatest influence on GGT with a weight of 26.13%.

### 3.5 Subgroup analysis

**Figure 4:**
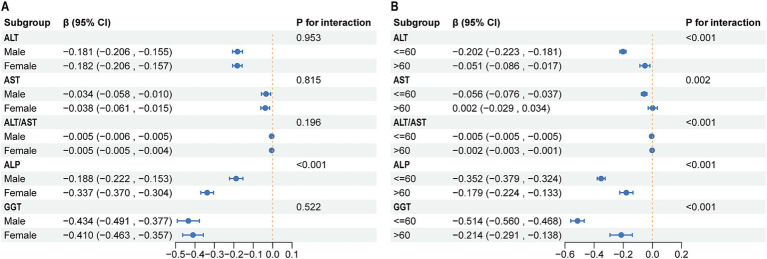
Subgroup analysis of the association between LE8 and liver function parameters. **(A)** Subgroup analyses stratified by sex. **(B)** Subgroup analyses stratified by sex. LE8, life’s Essential 8; ALT, alanine aminotransferase; AST, aspartate aminotransferase; GGT, gamma-glutamyl transferase; ALP, alkaline phosphatase.

In subgroup analyses stratified by sex, results showed consistent negative correlations between outcome variables (ALT, AST, ALT/AST, ALP, and GGT) and LE8 scores in both the male and female groups (Figure 4A). Specifically, the effect estimates for ALT were − 0.181 (95% CI: −0.206, −0.155) in males and − 0.182 (95% CI: −0.206, −0.157) in females, with no significant interaction between sex and ALT levels (*P* for interaction = 0.953). Similar patterns were observed for AST, ALT/AST ratio, ALP, and GGT, with no significant interaction effects except for ALP (*P* for interaction <0.001), where the effect was significantly stronger in females. Age-stratified analysis revealed notable differences in the associations (Figure 4B). For ALT, the effect estimate was stronger in participants aged 60 years or younger (−0.202, 95% CI: −0.223, −0.181) compared to those older than 60 years (−0.051, 95% CI: −0.086, −0.017), with a significant interaction between age and ALT levels (*P* for interaction <0.001). This interaction was also significant for AST, ALT/AST ratio, ALP, and GGT, indicating that the associations were modified by age. The effects were consistently more pronounced in the younger age group for most biomarkers, particularly for GGT.

## 4 Discussion

In this large cross-sectional study, we observed significant inverse associations between the LE8 score and liver function parameters, including ALT, AST, ALP, GGT, and the ALT/AST ratio. The relationship between LE8 scores and ALT and ALT/AST ratio showed nonlinear patterns, with significant decreases occurring at LE8 scores above 50.625 and 61.875, respectively. These findings underline the potential utility of the LE8 score in liver health monitoring, particularly for early identification of individuals at higher risk of liver dysfunction.

One of the key findings of this study was that the LE8 score was significantly associated with liver function parameters. A cross-sectional study by Labayen et al. recruiting 637 adolescents in 9 European countries found a positive association between ideal CVH and lower GGT and ALT (13). In a separate cross-sectional study of 1,084 European adolescents, they found that a lower AST/ALT ratio was also associated with higher cardiometabolic risk factors (16). A landmark study in 1995 first identified a significant association between GGT levels and mortality from coronary heart disease (17). Recent systematic reviews and meta-analyses have confirmed this association, showing a 60% increased relative risk of all-cause mortality in the highest tertile of GGT levels and a 7% increased risk per 5 U/L increase in GGT levels (18). In a cohort study of Austrian adults, high GGT was found to be significantly associated with CVD mortality in a dose–response relationship (19). In men and women, the hazard ratios for GGT were 1.66 and 1.64, respectively, with a stronger association in younger participants. In addition, the Rotterdam Study found that individuals in the top 5% of GGT levels had a 55% higher risk of all-cause mortality (20). Another meta-analysis showed a 56% increase in all-cause mortality for the highest versus lowest GGT quartile (21). Our study shows a negative association between LE8 scores and GGT levels, suggesting that better CVH, as reflected by higher LE8 scores, is associated with lower GGT levels. This finding is consistent with previous research and supports the role of GGT as a potential biomarker of CVH and mortality risk. The relationship between serum aminotransferases, particularly ALT and AST, with CVD risk has been extensively studied, though with varying degrees of association. However, when considering the LE8 score, which is designed to assess CVH, the interaction between these liver enzymes and LE8 components requires careful interpretation. Existing evidence suggests that the association between ALT and CVD risk is not as strong or consistent as that observed for GGT. For example, while the Framingham Offspring Heart Study found that elevated ALT levels were initially associated with a higher risk of CVD events, this association was attenuated after adjustment for multiple variables, suggesting that ALT may not independently predict CVD risk (22). However, an independent association between ALT levels and increased CVD mortality was found in a cohort study of 37,085 Korean participants (23). This nuanced relationship may extend to its association with LE8 scores, where ALT might correlate with some LE8 components, such as BMI and blood lipids, but not necessarily with overall cardiovascular risk. Similar trends are observed with AST, where its association with CVD events remains inconclusive. A meta-analysis of prospective cohort studies found no significant link between AST levels and increased risk of CVD mortality (24). Our results suggest a weak relationship between AST and LE8 score, which may reflect the limited role of CVH as an independent marker of AST. In contrast, ALP has shown a more consistent association with CVD risk. Higher levels of ALP have been associated with an increased risk of CHD and all-cause mortality, even after adjusting for traditional risk factors and excluding individuals with chronic kidney disease (25). This consistent association suggests that ALP may have a more direct relationship with CVH and, by extension, LE8 scores. The LE8 score, which includes several CVH factors, may interact with ALP levels in a way that reflects the enzyme’s role in vascular calcification and other cardiovascular processes.

The inverse relationship between the LE8 score and liver enzyme levels, particularly GGT, may be explained by several potential mechanisms. A healthier lifestyle, as reflected by a higher LE8 score, may reduce inflammatory stress, improve insulin sensitivity, and prevent excess adiposity (14). These factors contribute to a more favorable cardiovascular risk profile and may also reduce pathways leading to liver enzyme elevation. For example, adherence to dietary patterns such as the Mediterranean or DASH diets, which are characterized by a high intake of monounsaturated fatty acids, phytochemicals, fiber, and antioxidants, has been demonstrated to reduce inflammation and improve insulin resistance (26, 27). Participants who performed physical activity improved insulin sensitivity by decreasing immune cell activation and increasing glucose transporter type 4 translocation (28, 29). In addition, recent evidence indicates physical activity can directly affect lipogenesis and/or hepatic oxidation, thereby affecting hepatic lipid content (30). Avoiding obesity plays a critical role in preventing the release of inflammatory cytokines and free fatty acids from dysfunctional adipose tissue, which are known to contribute to lipotoxicity and hepatic steatosis (13, 31). Moreover, GGT has been implicated in promoting the oxidation of low-density lipoprotein (LDL) through redox reactions within atherosclerotic plaques, contributing to plaque development and progression (32). These mechanistic insights suggest that GGT is more strongly associated with LE8 scores than other liver enzymes, such as ALT, AST, and ALP, which may be due to its multifaceted role in oxidative stress and inflammation.

A key takeaway from this study is that the LE8 score can serve as an integrated measure for monitoring liver health and guiding CVH promotion. Given that CVD and liver dysfunction are often interconnected and share common risk factors, the LE8 score could serve as a dual marker to assess the overall health status of patients. Integrating LE8 scoring into routine clinical practice could be particularly beneficial in identifying individuals at high risk for both cardiovascular and liver diseases. By providing a holistic assessment of lifestyle factors, LE8 scores can help clinicians screen for early signs of liver dysfunction, such as elevated liver enzymes, while simultaneously monitoring cardiovascular risk. Furthermore, the use of the LE8 score in clinical practice could enhance personalized treatment strategies. For example, clinicians could tailor interventions to improve both cardiovascular and liver health based on a patient’s LE8 score. Interventions could include lifestyle modifications such as improved diet, increased physical activity, and smoking cessation. In this way, the LE8 score could contribute to a more integrated approach to managing patients’ overall health, potentially reducing the burden of both CVD and liver disorders. However, the feasibility of incorporating LE8 scoring into routine clinical practice would depend on the availability of relevant data in electronic health records and the development of standardized assessment tools for LE8 scoring. Training healthcare providers to interpret LE8 scores and use them to guide clinical decisions would also be necessary. Therefore, we call for future guidelines to consider incorporating the LE8 scores as part of routine health assessments to better understand its impact on patient outcomes and healthcare efficiency.

The strengths of our study are noteworthy. A key strength is the innovative use of the LE8 score, a comprehensive metric that integrates multiple lifestyle factors to provide a holistic assessment of CVH. This comprehensive approach may provide valuable insights into identifying individuals at higher risk for liver function abnormalities who may benefit from targeted interventions. In addition, our study used the WQS regression model, a novel methodological approach that allowed us to identify the most influential components of the LE8 score on liver enzyme levels. The use of data from the NHANES, a large-scale, nationally representative cross-sectional survey, further strengthens the generalizability of our findings to the broader U.S. population. The multistage probability sampling design of NHANES ensures that our results are applicable to different demographic groups. Furthermore, our study included detailed subgroup and interaction analyses, which provided a deeper understanding of how different population characteristics may influence the relationship between the LE8 score and liver enzyme levels. This approach highlights the necessity of adapting interventions to particular subgroups, thereby increasing the likelihood of developing more personalized and effective prevention strategies.

This study has several limitations. First, the cross-sectional design of the study limits the ability to infer causality. Although we observed correlations between LE8 scores and liver function, causal relationships cannot be established. Second, despite adjustment for numerous potential confounders, it is not possible to completely eliminate all sources of bias. For example, dietary recall data based on 24-h recall methods may be susceptible to recall or reporting bias, potentially affecting the accuracy of dietary intake data. Finally, because the NHANES database does not provide exact dates for dietary recall interviews and blood sample collection, we are unable to directly analyze the temporal relationship between these variables, which limits the assessment of time-dependent effects of dietary intake and blood biomarkers on liver function parameters.

## 5 Conclusion

The present study reveals a significant inverse relationship between the LE8 scores and liver enzyme levels. This finding indicates that higher LE8 scores, which reflect better CVH, are associated with improved liver function. Nonlinear analyses identified key inflection points for ALT and the ALT/AST ratio, indicating that the advantages of elevated LE8 scores on liver function may be more pronounced above specific thresholds. Given the potential of the LE8 score to guide early identification of individuals at risk for liver diseases, future guidelines could incorporate the LE8 score as part of routine screening and preventive measures. However, given the limitations of the current study, future prospective studies are needed to confirm these associations and explore the underlying mechanisms further.

## Data Availability

The original contributions presented in the study are included in the article/Supplementary material, further inquiries can be directed to the corresponding author.
